# Predictive factors of posttreatment fracture by definitive radiotherapy for uterine cervical cancer

**DOI:** 10.1007/s11604-020-01039-8

**Published:** 2020-09-07

**Authors:** Kazuki Ishikawa, Tsuneo Yamashiro, Takuro Ariga, Takafumi Toita, Wataru Kudaka, Joichi Heianna, Hitoshi Maemoto, Takeaki Kusada, Wataru Makino, Yoichi Aoki, Sadayuki Murayama

**Affiliations:** 1 Department of Radiology, Graduate School of Medical Science, University of the Ryukyus, 207 Uehara, Nishihara, Okinawa 903-0215 Japan; 2 Health Information Management Center, University of the Ryukyus Hospital, 207 Uehara, Nishihara, Okinawa 903-0215 Japan; 3 Radiation Therapy Center, Okinawa Chubu Hospital, 281 Miyazato, Uruma, Okinawa 904-2293 Japan; 4 Department of Obstetrics and Gynecology, Graduate School of Medical Science, University of the Ryukyus, 207 Uehara, Nishihara, Okinawa 903-0215 Japan

**Keywords:** Fracture, Cervical cancer, Radiotherapy, Computed tomography, CT densities, Uterus

## Abstract

**Purpose:**

Fractures are known to shorten life expectancy and worsen the quality of life. The risk of fractures after radiation therapy in cervical cancer patients is known to be multifactorial. In this study, we examined risk factors for fractures in cervical cancer patients, especially by evaluating bone densities and DVH parameters for fractured bones.

**Materials and Methods:**

For 42 patients, clinical characteristics, pretreatment CT bone densities, and radiation dose were compared between patients with and without fractures.

**Results:**

Posttreatment fractures occurred in 25 bones among ten patients. Pretreatment CT bone densities were significantly lower in patients with fractures (*P* < 0.05–0.01 across sites, except for the ilium and the ischium). Although DVH parameters were also significantly associated with fractures in univariate analysis, only CT densities were significantly associated with fractures in multivariate analysis.

**Conclusion:**

Pretreatment CT densities of spinal and pelvic bones, which may reflect osteoporosis, have a significant impact on the risk for posttreatment fractures.

**Electronic supplementary material:**

The online version of this article (10.1007/s11604-020-01039-8) contains supplementary material, which is available to authorized users.

## Introduction

Definitive radiotherapy (dRT) for uterine cervical cancer is a standard treatment at Fédération Internationale de Gynécologie et d'Obstétrique [FIGO] 2008 stage IB toIVA, and a common treatment option for elderly women. Recent studies have revealed that pelvic fractures—including insufficiency fractures— occur in 10–29% of patients [1–10]. Fractures are known to shorten life expectancy and worsen the quality of life [11, 12], so it is necessary to identify risk factors to prevent fractures. Several reports have evaluated considerable risk factors for fracture, such as advanced age [1–3, 5, 8], osteoporosis [8], low body weight/body mass index (BMI) [1, 2, 7], postmenopausal status [2, 5, 8], radiation dosage [3], brachytherapy [9], and chemotherapy [3].

Some reports indicate that pretreatment CT densities, which may reflect osteoporosis, ​​may be a risk indicator for fractures [8, 13]. As CT scans only evaluate parts of the bones, they may be different depending on the location of the measurement areas.

A recent study indicated that reducing pelvic bone radiation doses decreased the risk of pelvic fracture [14]. Some reports have analyzed the radiation dose distribution of pelvic bones without calculating the radiation exposure from intracavitary brachytherapy (ICBT) [8, 10]. As the position of the uterus may change during the RT procedure [15], the cumulative dose of each ICBT needs to be properly calculated. Also, no previous report has evaluated the radiation dose contributed by combined whole-pelvic external beam radiotherapy (WPRT) with the midline-block (MB) technique, which is commonly performed in Japan, and ICBT which is used to assess the risk of pelvic fracture. Furthermore, previous studies did not evaluate cumulative doses separately for each pelvic bone [16]. It is necessary to measure each bone separately because each bone may have different risk factors.

In this study, we examined which factors are suitable for predicting the fracture of the lumbar spine and pelvic bones, and we looked for dose volume histogram (DVH) parameters to predict fractures.

### Materials and methods

The Institutional Review Board of our institution approved this retrospective study. The need for written informed consent from the patients was waived by the Board.

### Patients and treatment details

A total of 66 patients with cervical cancer underwent dRT or concurrent chemoradiotherapy (CCRT) between October 2011 and December 2013. Among them, 15 patients were ineligible to participate in the study because they underwent extended-field RT, four patients were excluded as a result of discontinuing RT, and the other five patients were excluded because diagnostic computed tomography (CT) had not been performed after RT. Finally, 42 patients were eligible for the study and subsequently enrolled. Twenty-nine patients were treated with CCRT, and 13 patients underwent dRT alone. The CCRT regimen was weekly cisplatin (CDDP) or weekly CDDP with paclitaxel (TP).

Patients received WPRT prior to high-dose-rate (HDR) ICBT. An initial dose of radiation, 40 Gy in 20 fractions, was delivered using a 4-field box technique; subsequently, 10 Gy in 5 fractions was delivered with a 4-cm-wide MB through anteroposterior portals. For the 14 patients who had pelvic lymph node metastasis or parametrial infiltration, a boost of 6 Gy in 3 fractions was delivered.

All patients received ICBT via tandem and ovoid applicators. ICBT was delivered using an HDR ^192^Ir remote after-loading system (RALS) (microSelectron, Elekta, Stockholm, Sweden). The dose prescribed to point A with 18 Gy in 3 fractions was selected according to the Manchester System. A 4-row multidetector CT scanner (LightSpeed-RT, GE Healthcare, Milwaukee, WI, USA) was used for CT simulations with a 2.5-mm slice thickness. All ICBT was simulated and performed using the 3D-CT scan image-guided brachytherapy (IGBT). All CT simulations for WPRT, the boost, and each ICBT were performed using the same CT scanner.

### Image analysis for bone fracture, structure contouring, and measuring CT densities

All 42 patients underwent a diagnostic CT before dRT, which is different from a simulation CT. After dRT, CT scans were taken every three to six months for monitoring purposes following treatment for cervical cancer, as well as lymph node and visceral metastases. A total of 263 diagnostic CT images were acquired (3–11 scans per patient).

By comparing pretreatment with posttreatment CT scans, new fractures of the lumbar spine (L4 and L5) and pelvic bones were recorded and assessed by an experienced, board-certified, diagnostic radiologist of the Japan Radiological Society. The definition of fractures includes an apparent fracture line, sclerotic change, or deformation of bone, such as a compression fracture. Any bone fractures that were identified were confirmed by two cooperating radiation oncologists. None of the 42 patients had a fracture attributable to bone metastasis or trauma based on a review of the medical records.

**Fig. 1:**
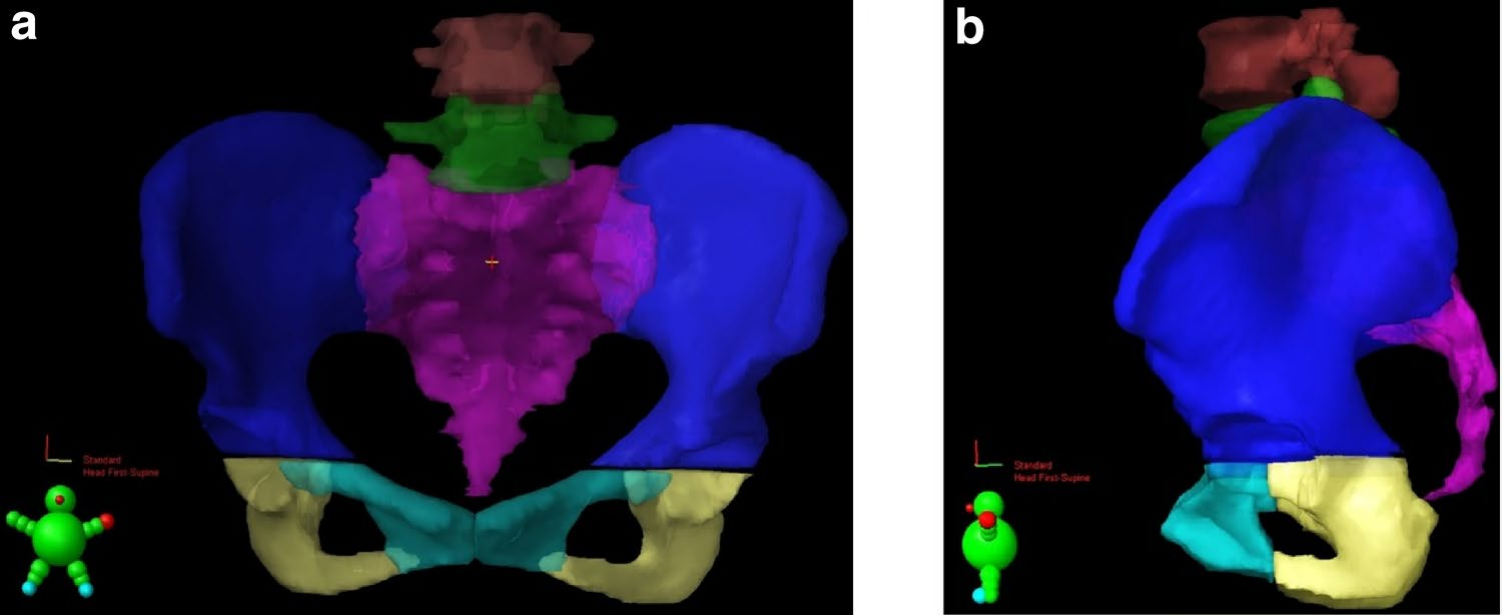
Bone contour: pelvic bones divided based on the center of the femoral head, the cranial side of the pelvic bone was defined as the ilium, the inferior-anterior side as the pubic bone, and the inferior-posterior aspect as the ischium. **a** Front view. **b** Side view

All simulation CTs were taken on the same CT machine without using contrast media, and one radiation oncologist (K.I.) contoured the following nine bones: L4, L5, sacrum, ilium (right and left), ischium (right and left), and pubis (right and left). Based on the center of the femoral head, the cranial side of the pelvic bone was defined as the ilium, the inferior-anterior side as the pubic bone, and the inferior-posterior aspect as the ischium (Fig. 1). A senior radiation oncologist (T.A.) confirmed that the bones were correctly contoured. Using the contoured bones on the simulation CT scans, the average Hounsfield Unit (HU) values of the nine bones were measured automatically.

### Accumulation of RT dose

**Fig. 2:**
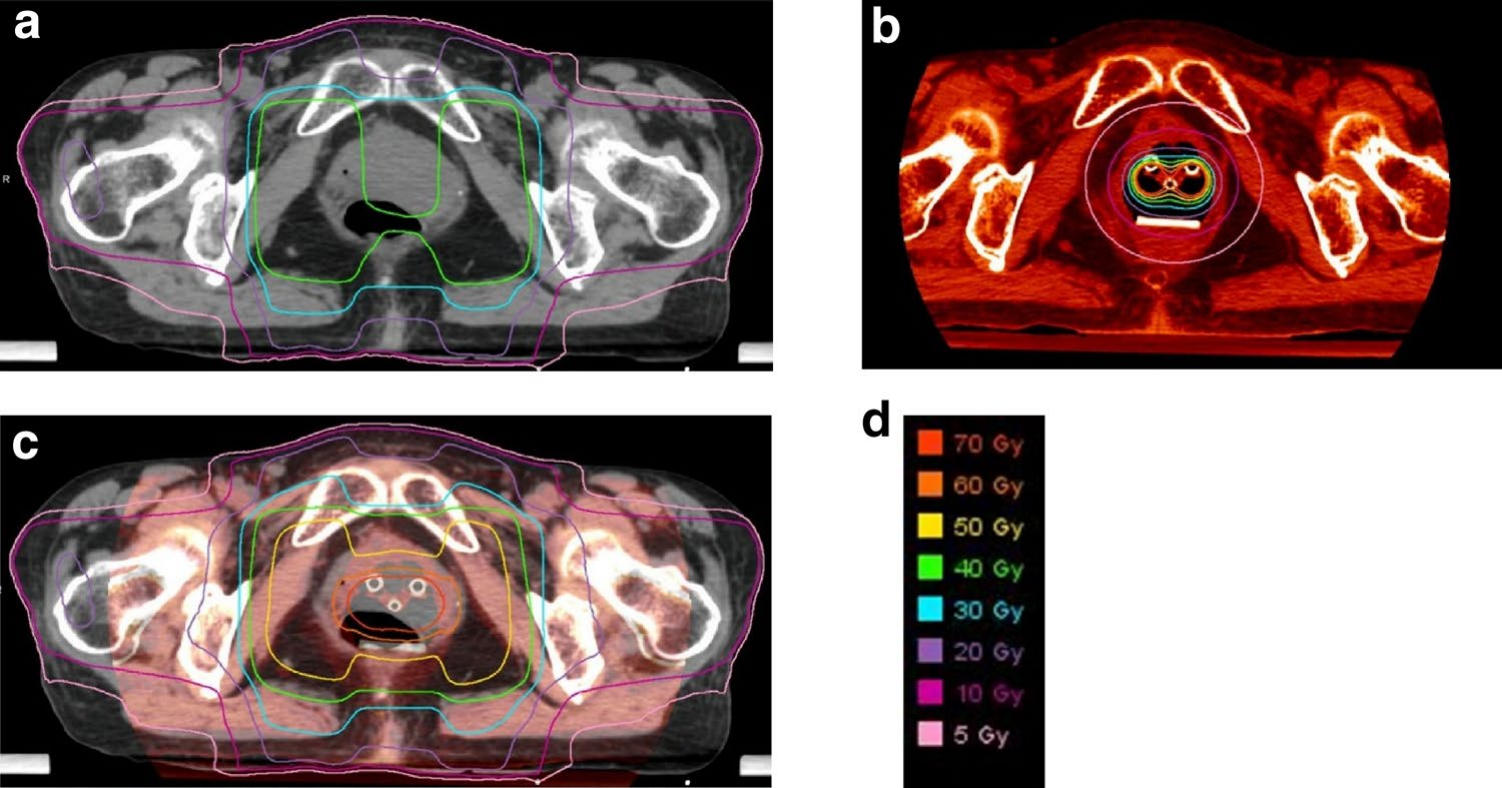
Dose distribution: **a** Whole pelvic radiotherapy with 4-field box technique and with midline block, **b** all intracavitary brachytherapy dose, **c** accumulation dose of Fig. 2a, b. **d** Corresponding dose and colors

We used MIM Maestro software (MIM software, OH, USA) for converting the cumulative doses to an equivalent dose in 2 Gy (EQD2) for both EBRT and ICBT using a linear-quadratic model with α/β of 3 for the pelvic bones. One radiation oncologist (K.I.) accumulated RT dose. First, the dose of each ICBT was converted into an EQD2 and accumulated using a bone-matched rigid fusion method on CT images for simulation of the first-time ICBT. Second, all doses of the ICBT were transferred on the CT images for the simulation of WPRT using a bone-matched rigid fusion method, and the cumulative doses of WPRT (using the MB technique) and ICBT were measured (Fig. 2). Then, DVH parameters, including V10 to 50 Gy (every 10 Gy), D2cc, and the mean dose delivered to each bone, was calculated.

### Statistical analyses

The effects of patient characteristics (e.g., age, height, weight, BMI, menopause status, pathology, FIGO 2008 stage, and individual bone CT densities) were assessed using the Mann–Whitney test and Chi-square test. The threshold values of CT density for detecting fractures were calculated using the Youden Index of the ROC curve. The effect of the dose of radiation delivered on fracture incidence was estimated using the Kaplan–Meier method. The Cox regression model was used for multivariate analysis. These all analyses were performed using JMP version 15.0.0 (SAS Institute, NC, USA). *P* values < 0.05 were considered statistically significant for all analyses.

## Results

### Fracture incidence

The median CT scan follow-up period from the first day of RT was 47 months (range 8–68 months). Fractures occurred in 25 bones among ten patients (23.8%), and the median time interval from dRT initiation to fracture detection was 14 months (range 4–41 months). Eight people sustained multiple fractures at two or more locations (two fractures at different locations in five patients, three fractures in two patients, and five fractures in one patient). Fractures were sustained by patients at various sites as follows: L4, *n* = 6 (14.2%); L5, *n* = 4 (9.5%); sacrum, *n* = 7 (16.7%); ilium, *n* = 4 (9.5%) (bilateral fractures, *n* = 1); and pubis, *n* = 2 (4.8%) (bilateral fractures, *n* = 1). No patients sustained an ischium fracture. All fractured bones were included within the irradiated volumes.

### Patient characteristics and fracture rate

The characteristics of the 42 patients are summarized in Table 1. There was no significant difference in the overall survival rate for patients with or without fractures (without fracture patients, 92%; with fracture patients, 81% survival rate at 47 months; *P* = 0.42).

**Table 1:** Patient characteristics

	Patients (*n* = 42)	Mean ± SD
Age (years)		57.5 ± 15.1
BMI (kg/m^2^)		23.3 ± 5.4
Chemotherapy		
Yes	29	
No	13	
FIGO 2008 stage		
IB1	10	
IB2	6	
IIA	1	
IIB	13	
IIIA	0	
IIIB	12	
Menopause		
Yes	22	
No	20	
Pathology		
SCC	37	
Adenocarcinoma	5	

*BMI* body mass index; *FIGO* Fédération Internationale de Gynécologie et d'Obstétrique; *SCC* squamous cell carcinoma

The relationships between each bone site fractured and patient characteristics are summarized in Table 2. There was a more highly significant relationship between L4 and L5 fractures among patients of advanced age (≥ 65 years) than among patients of a younger age (*P* < 0.05). Fractures at L4 and the pubis were more frequent when patients had a low BMI (< 20 kg/m^2^; *P* < 0.05). L4 and L5 fractures were more frequently observed among patients who did not use concomitant anticancer drugs than among patients prescribed anticancer drugs (*P* < 0.05 and *P* < 0.01, respectively). Among postmenopausal patients, L5 fractures were more commonly observed than they were among premenopausal patients (*P* < 0.05).

**Table 2:** Fractures of the spine and pelvic bones with patient characteristics (*n* = 42)

	L4			L5			Sacrum			Ilium			Pubis			Ischium*	
	Fracture ( +)	Fracture (−)	*P* value	Fracture ( +)	Fracture (−)	*P* value	Fracture ( +)	Fracture (−)	*P* value	Fracture ( +)	Fracture (−)	*P* value	Fracture ( +)	Fracture (−)	*P* value	Fracture ( +)	Fracture (−)
Patient (n = 42)	6 (14.2%)	36 (85.8%)		4 (9.5%)	38 (90.5%)		7 (16.7%)	35 (83.3%)		4 (9.5%)	38 (90.5%)		2 (4.8%)	40 (95.2%)		0 (0%)	42 (100%)
Age≧65			< 0.05			< 0.01			NS			NS			NS		
Yes	4	9		4	9		3	10		1	12		1	12			
No	2	27		0	29		4	25		3	26		1	28			
BMI < 20			< 0.05			NS			NS			NS			< 0.05		
Yes	4	8		2	10		2	10		1	11		2	10			
No	2	28		2	28		5	25		3	27		0	30			
Chemotherapy			< 0.05			< 0.01			NS			NS			NS		
Yes	2	27		0	29		4	25		3	26		1	28			
No	4	9		4	9		3	10		1	12		1	12			
FIGO 2008 stage			NS			NS			NS			NS			NS		
IIA or less	1	16		1	16		1	16		1	16		0	17			
IIB or greater	5	20		3	22		6	19		3	22		2	23			
Menopause			NS			< 0.05			NS			NS			NS		
Yes	5	17		4	18		5	17		3	19		2	20			
No	1	19		0	20		2	18		1	19		0	20			
Pathology			NS			NS			NS			< 0.05			NS		
SCC	6	31		4	33		5	32		2	35		1	36			
Adenocarcinoma	0	5		0	5		2	3		2	3		1	4			

*L* lumbar spine, *BMI* body mass index, *FIGO* Fédération Internationale de Gynécologie et d'Obstétrique, *SCC* squamous cell carcinoma, *NS* not significant

*Since no fracture was found in the ischium, statistical analysis was not performed

## CT bone density and fracture

Table 3 shows differences in the CT densities between patients with and without a fracture at the sites examined. Overall, CT densities were significantly lower among patients with fractures (L4:* P* < 0.05, L5: *P* < 0.05, sacrum: *P* < 0.05, pubis: *P* < 0.01), except at the ilium and the ischium.

**Table 3:** Average CT densities of the spine and pelvic bones with and without fracture

Bone	Fracture ( +)	Fracture (−)	*P* value	Cut-off
L4	*n* = 6	*n* = 36		
CT density (HU)	254.8 ± 67.7	322.5 ± 61.8	< 0.05	256.5
L5	*n* = 4	*n* = 38		
CT density (HU)	213.2 ± 47.9	329.5 ± 53.3	< 0.01	259.5
Sacrum	*n* = 7	*n* = 35		
CT density (HU)	182.5 ± 44.3	232.9 ± 60.1	< 0.05	244.3
Ilium*	*n* = 5	*n* = 79		
CT density (HU)	276.6 ± 78.5	342.6 ± 74.2	0.07	
Pubis*	*n* = 3	*n* = 81		
CT density (HU)	186.6 ± 31.6	278.4 ± 51.6	< 0.01	222.2

*L* lumbar spine, *CT* computed tomography, *HU* Hounsfield Unit

Asterisk indicates that the right and left bones were analyzed in a single statistical model

### Dose distribution and fracture

Fractures of the pubis were more frequently identified in the group of patients exposed to a higher amount of radiation than in the groups with lower exposures as follows: V30 Gy > 75% or ≦75%, *P* < 0.05; V40 Gy > 55% or ≦55%, *P* < 0.01; and V50 Gy > 25% or ≦25%, *P* < 0.05. This trend was not evident in relation to the fractures of other bones (Supplementary material).

### Multivariate analysis

Multivariate analysis was performed on factors that were significant in univariate analyses. Among these factors, advanced age, low BMI, and postmenopause status were found to be correlated with CT densities. Therefore, multivariate analysis was constructed using CT densities and DVH parameters (V30 Gy, V40 Gy, and V50 Gy) for pubic fractures. Finally, only the CT densities were a significant factor (*P* < 0.001) for pubic bone fractures.

## Discussion

Pretreatment CT densities of spinal and pelvic bones had a significant impact on the risk of posttreatment fractures. In previous studies, several factors have been identified that have a significant effect on fractures of the lumbar spine and pelvic bones. Low bone mineral density (BMD) is one of the most highly predictive factors for fractures because it reflects osteoporosis [17]. However, BMD measurement is not routinely performed during standard clinical care for cervical cancer. In fact, BMD data had not been collected previously for almost all patients in our study cohort. Furthermore, since BMD is usually calculated from sites, such as the lumbar spine and femoral neck, it is difficult to evaluate pathologic bone mineral loss in the specific bones that are affected by dRT in patients with cervical cancer. Thus, planning to measure bone density using CT before dRT may be useful in predicting the risk of a subsequent fracture. Compared with a previous study, during which bone density was measured using CT and manually selecting a focal region of interest within the measured bone [8], we contoured the bone cortex and medulla of all targeted bones of interest. Considering that almost all spinal and pelvic bones with a lower BMD assessed using CT exhibited posttreatment fractures more frequently than bones with a higher BMD, we regard the approach that we have outlined in this study as potentially useful to predict the risk of fractures post-RT in affected bones. We could show that pretreatment CT densities of spinal and pelvic bones, which may reflect osteoporosis, are useful for predicting fracture. Still, future studies are recommended to determine the most convenient and efficient method by which to plan to evaluate osteoporosis using CT scans in this patient population.

Exposure to higher amounts of radiation via dRT used for the treatment of cervical cancer is considered as a risk factor for fracture [3, 16]. However, few studies have examined DVH of accumulated doses of WPRT and ICBT. Although Ramlov et al. investigated the accumulated doses of EBRT and ICBT [16], no study had indicated DVH parameters in Japanese studies, which usually treated with MB technique [1, 5, 7–10]. Therefore, we accumulated all RT dose and analyzed the relation between DVH parameters and pelvic bone fractures. We reported that there was significant p-value in the result of the Kaplan–Meier method between the pubic bone fracture and some DVH parameters, such as V30 Gy > 75%, V40 Gy > 55%, and V50 Gy > 25%. Considering that the number of pubic bone fracture was only three locations in two patients, and such DVH parameters were not indicated in more fractured bones, we had to interpret the results carefully.

We performed multivariate analysis for CT densities ​​and DVH parameters (V30 Gy, V40 Gy, and V50 Gy), except for advanced age, BMI, and postmenopause status, which showed a significant difference in univariate analysis. Since advanced age, low BMI, and postmenopause status are associated with BMD, they are confounding factors of CT densities. As a result, DVH parameters were not a significant factor in multivariate analysis. We finally concluded that there was no significant relation between DVH parameter and pubic bone fracture. From these results of this study, we thought that CT densities are the most contributed factor for the risk of fracture.

Similar to previous studies that have shown advanced age, low body weight, and menopause as predictive factors for fracture [1–9], L4, L5, and pubic fractures were significantly associated with age, BMI, and menopause in this study. This suggests that our study population can be considered similar to the previous populations. In contrast, patients in the current study who had received chemotherapy tended to exhibit fractures less frequently than patients who did not receive chemotherapy. This could be because patients treated with chemotherapy were younger and in better physical condition, and it is predicted that they were the more bone-dense group. This proposal should be further evaluated in future studies to determine whether treatment with anticancer drugs can reduce the risk of fractures post-RT.

Previous studies have reported that fractures reduce QOL and survival [11, 12], but this study found no significant effect of the presence or absence of fractures on the overall survival rate (patients without fracture, 92% survival rate at 47 months; patients with fracture, 81%. *P* = 0.42). This may be due to the small number of patients with fractures and the short follow-up period.

Our study has several limitations: First, since this study was retrospective, available patient information was limited. Second, the total number of enrolled patients was small, resulting in a low number of fractures in the bones targeted for observation. Third, because the follow-up observation protocol was not standardized at our institution, there was a wide range in the observation period for the posttreatment CT scans. This may have resulted in an underestimation of the number of fractures, particularly among those patients whose follow-up period was short. Fourth, enrolled patients could have harbored slight or undetectable fractures, considering that magnetic resonance imaging or bone scintigraphy was not routinely used to evaluate for existing fractures.

In conclusion, pretreatment CT densities of the spinal and pelvic bones had a significant impact on fractures; furthermore, in univariate analysis, advanced age, low BMI, postmenopause status, and DVH parameters were also significantly associated with several bone fractures. However, the CT densities were the only significant factor in the multivariate analysis. In the future, we will investigate whether these factors, which were pointed out in the univariate analysis, are statistically significant or not in cases with CT densities adjusted patient groups.

## Electronic supplementary material

Below is the link to the electronic supplementary material.

Supplementary file1 (DOCX 37 kb)
