# Supplementary material for: Predictive factors of posttreatment fracture by definitive radiotherapy for uterine cervical cancer
Source: Jpn J Radiol. 2020 Sep 7;39(1):93–9. doi: 10.1007/s11604-020-01039-8 (PMC7813741; doi:10.1007/s11604-020-01039-8)
Supplement: Supplementary file 1 — Supplementary file1 (DOCX 37 kb) [file 11604_2020_1039_MOESM1_ESM.docx]

**Supplementary material.**

The result of the Kaplan-Meier analysis of dose volume histograms on lumber and pelvic bone fracture.

| Pubis  (Fx = 3/84) | |  |  |  |
| --- | --- | --- | --- | --- |
|  |  | Yes | No | P-value |
| V30 | >40%  >45%  >50%  >55%  >60% | 74(2)  70(2)  68(2)  64(2)  57(2) | 10(1)  14(1)  16(1)  20(1)  27(1) | 0.33  0.55  0.65  0.84  0.89 |
|  | >65% | 50(2) | 34(1) | 0.64 |
|  | >70% | 47(2) | 37(1) | 0.29 |
|  | >75% | 21(2) | 63(1) | <0.05 |
|  | >80% | 12(2) | 72(1) | <0.001 |
| V40 | >20%  >25%  >30%  >35%  >40% | 76(2)  71(2)  66(2)  57(2)  49(2) | 8(1)  13(1)  18(1)  27(1)  35(1) | 0.22  0.50  0.74  0.89  0.61 |
|  | >45% | 41(2) | 43(1) | 0.40 |
|  | >50% | 32(2) | 52(1) | 0.65 |
|  | >55% | 15(2) | 69(1) | <0.01 |
|  | >60% | 9(1) | 75(2) | <0.04 |
| V50 | >5%  >10% | 72(2)  59(2) | 12(1)  25(1) | 0.44  0.96 |
|  | >15% | 46(2) | 38(1) | 0.51 |
|  | >20% | 49(2) | 35(1) | 0.24 |
|  | >25% | 23(2) | 61(1) | <0.05 |
|  | >30% | 12(1) | 72(2) | 0.12 |
|  | >35%  >40%  >45-50%  >50-95% | 7(1)  2(0)  1(0)  0(0) | 77(2)  82(3)  83(3)  84(3) | <0.02  -  -  - |

| L4  (Fx = 6/42) | |  |  |  |
| --- | --- | --- | --- | --- |
|  |  | Yes(Fx) | No(Fx) | P-value |
| V30 | >40-45% | 13(3) | 29(3) | 0.42 |
|  | >50% | 10(3) | 32(3) | 0.24 |
|  | >55%  >60%  >65%  >70-75%  >80% | 8(3)  7(3)  6(2)  4(1)  3(1) | 34(3)  35(3)  36(4)  38(5)  39(5) | 0.09  0.08  0.50  0.41  0.21 |
| V40 | >20% | 17(3) | 25(3) | 0.74 |
|  | >25% | 15(3) | 27(3) | 0.53 |
|  | >30% | 14(3) | 28(3) | 0.44 |
|  | >35% | 13(3) | 29(3) | 0.42 |
|  | >40% | 10(3) | 32(3) | 0.24 |
|  | >45%  >50%  >55%  >60% | 8(3)  6(2)  4(1)  3(1) | 34(3)  36(4)  38(5)  39(5) | 0.09  0.50  0.38  0.21- |
| V50 | >5%  >10%  >15%  >20-40% | 5(1)  4(1)  3(1)  1(0) | 37(5)  38(5)  39(5)  41(6) | 0.42  0.24  0.21  - |

| L5  (Fx = 4/42) | |  |  |  |
| --- | --- | --- | --- | --- |
|  |  | Yes(Fx) | No(Fx) | P-value |
| V30 | >40-45%  >50-55%  >60%  >65%  >70% | 38(3)  37(3)  36(2)  38(3)  32(2) | 4(1)  5(1)  6(2)  4(1)  10(2) | 0.40  0.56  0.08  0.40  0.35 |
|  | >75% | 29(1) | 13(3) | 0.08 |
|  | >80% | 26(1) | 16(3) | 0.16 |
| V40 | >20%  >25-40%  >45%  >50%  >55%  >60% | 39(4)  38(3)  37(3)  36(2)  35(2)  33(2) | 3(0)  4(1)  5(1)  6(2)  7(2)  9(2) | -  0.40  0.56  0.08  0.14  0.28 |
| V50 | >5%  >10% | 31(2)  28(2) | 11(2)  14(2) | 0.32  0.56 |
|  | >15-20% | 24(2) | 18(2) | 0.89 |
|  | >25%  >30%  >35%  >40% | 21(0)  18(0)  15(0)  13(0) | 21(4)  24(4)  27(4)  29(4) | -  -  -  - |

| Sacram  (Fx = 7/42) | |  |  |  |
| --- | --- | --- | --- | --- |
|  |  | Yes(Fx) | No(Fx) | P-value |
| V30 | >40%  >45-65%  >70-75%  >80% | 40(7)  39(6)  38(6)  29(4) | 2(0)  3(1)  4(1)  13(3) | -  0.30  0.57  0.35 |
| V40 | >20%  >25%  >30-40%  >45%  >50%  >55% | 41(7)  40(7)  39(6)  38(6)  37(6)  29(5) | (0)  2(0)  3(1)  4(1)  5(1)  13(2) | -  -  0.30  0.57  0.81  0.94 |
|  | >60% | 24(4) | 18(3) | 0.89 |
| V50 | >5%  >10%  >15%  >20%  >25% | 41(7)  39(7)  37(6)  35(6)  33(5) | 1(0)  3(0)  5(1)  7(1)  9(2) | -  -  0.74  0.88  0.61 |
|  | >30% | 26(5) | 16(2) | 0.49 |
|  | >35% | 16(2) | 26(5) | 0.55 |
|  | >40% | 11(1) | 31(6) | 0.47 |

| ilium  (Fx = 5/84) | |  |  |  |
| --- | --- | --- | --- | --- |
|  |  | Yes(Fx) | No(Fx) | P-value |
| V30 | >40% | 57(3) | 27(2) | 0.54 |
|  | >45% | 30(2) | 54(3) | 1.00 |
|  | >50%  >55%  >60-65%  >70-80% | 9(0)  3(0)  1(0)  0(0) | 75(5)  81(5)  83(5)  84(5) | -  -  -  - |
| V40 | >20%  >25%  >30% | 84(5)  75(5)  62(5) | 0(0)  9(0)  22(0) | -  -  - |
|  | >35% | 27(1) | 57(4) | 0.46 |
|  | >40%  >45-50%  >55-60% | 6(0)  1(0)  0(0) | 78(5)  83(5)  84(5) | -  -  - |
| V50 | >5%  >10%  >15%  >20% | 84(5)  82(5)  74(5)  62(5) | 0(0)  0(0)  10(0)  22(0) | -  -  -  - |
|  | >25%  >30%  >35-40% | 23(1)  2(0)  1(0) | 61(4)  82(5)  83(5) | 0.65  -  - |

**Abbreviations:** Fx = fracture; V30 = the percentage of bone volume receiving > 30 Gy; V40 = the percentage of bone volume receiving > 40 Gy; V50 = the percentage of bone volume receiving > 50 Gy; NS = not significant
